# Protocol for an HIV Pre-exposure Prophylaxis (PrEP) Population Level Intervention Study in Victoria Australia: The PrEPX Study

**DOI:** 10.3389/fpubh.2018.00151

**Published:** 2018-05-29

**Authors:** Kathleen E. Ryan, Anne Mak, Mark Stoove, Brian Price, Christopher K. Fairley, Simon Ruth, Luxshimi Lal, Jason Asselin, Carol El-Hayek, Long Nguyen, Colin Batrouney, David Wilson, John Lockwood, Dean Murphy, Vincent J. Cornelisse, Norman Roth, Jeff Willcox, Christina C. Chang, Judy Armishaw, Ban K. Tee, Matthew Penn, George Forgan-Smith, Christopher Williams, Jeff Montgomery, Kat Byron, Alison Coelho, Brent Allen, Jeremy Wiggins, Jenny Kelsall, Olga Vujovic, Michael West, Anna B. Pierce, Daniel Gallant, Charlotte Bell, John B. F. de Wit, Jennifer F. Hoy, Steve L. Wesselingh, Robert M. Grant, Edwina J. Wright

**Affiliations:** ^1^The Burnet Institute, Melbourne, VIC, Australia; ^2^Department of Epidemiology and Preventive Medicine, Monash University, Melbourne, VIC, Australia; ^3^Alfred Health Pharmacy, Melbourne, VIC, Australia; ^4^Alfred Health, Melbourne, VIC, Australia; ^5^Melbourne Sexual Health Centre, Melbourne, VIC, Australia; ^6^Central Clinical School, Monash University, Melbourne, VIC, Australia; ^7^Victorian AIDS Council, Melbourne, VIC, Australia; ^8^Centre for Social Research in Health, University of New South Wales, Sydney, NSW, Australia; ^9^Prahran Market Clinic, Melbourne, VIC, Australia; ^10^Northside Clinic, Melbourne, VIC, Australia; ^11^Department of Infectious Diseases, Alfred Hospital and Monash University, Melbourne, VIC, Australia; ^12^The Centre Clinic, Melbourne, VIC, Australia; ^13^Access Health and Community Richmond, Melbourne, VIC, Australia; ^14^PRONTO! Community Health Centre, Melbourne, VIC, Australia; ^15^ERA Health, Melbourne, VIC, Australia; ^16^PrEP'DForChange, Melbourne, VIC, Australia; ^17^PrEPaccessNOW, Melbourne, VIC, Australia; ^18^Victorian Aboriginal Community Controlled Health Organisation Inc., Melbourne, VIC, Australia; ^19^Centre for Culture, Ethnicity and Health, Melbourne, VIC, Australia; ^20^Living Positive Victoria, Melbourne, VIC, Australia; ^21^Peer Advocacy Network for the Sexual Health of Trans Masculinities, Melbourne, VIC, Australia; ^22^Harm Reduction Victoria, Melbourne, VIC, Australia; ^23^Sexual Health and Viral Hepatitis Service Department of Health and Human Services, Government of Victoria, Melbourne, VIC, Australia; ^24^Sexually Transmissible Infection and Blood Borne Virus Section, Communicable Disease Control Branch, Department of Health and Ageing, Government of South Australia, Adelaide, SA, Australia; ^25^Clinic 275, Royal Adelaide Hospital, Adelaide, SA, Australia; ^26^South Australian Health and Medical Research Institute, Adelaide, SA, Australia; ^27^School of Medicine, University of California San Francisco, Gladstone Institutes, San Francisco, CA, United States; ^28^Peter Doherty Institute for Infection and Immunity, Melbourne, VIC, Australia

**Keywords:** PrEP, HIV, MSM, prevention, protocol

## Abstract

**Background:** Pre-exposure prophylaxis (PrEP) is the use of HIV anti-retroviral therapy to prevent HIV transmission in people at high risk of HIV acquisition. PrEP is highly efficacious when taken either daily, or in an on-demand schedule. In Australia co-formulated tenofovir-emtricitabine is registered for daily use for PrEP, however, this co-formulation is not listed yet on the national subsidized medicines list. We describe a study protocol that aims to demonstrate if the provision of PrEP to up to 3800 individuals at risk of HIV in Victoria, Australia reduces HIV incidence locally by 25% generally and 30% among GBM.

**Methods:** PrEPX is a population level intervention study in Victoria, Australia in which generic PrEP will be delivered to 3800 individuals for up to 36 months. Study eligibility is consistent with the recently updated 2017 Australian PrEP guidelines. Participants will attend study clinics, shared care clinics, or outreach clinics for quarterly HIV/STI screening, biannual renal function tests and other clinical care as required. Study visits and STI diagnoses will be recorded electronically through the ACCESS surveillance system. At each study visit participants will be invited to complete behavioral surveys that collect demographics and sexual risk data. Diagnosis and behavioral data will be compared between PrEPX participants and other individuals testing within the ACCESS surveillance system. A subset of participants will complete in depth surveys and interviews to collect attitudes, beliefs and acceptability data. Participating clinics will provide clinic level data on implementation and management of PrEPX participants. The population level impact on HIV incidence will be assessed using Victorian HIV notification data.

**Discussion:** This study will collect evidence on the real world impact of delivery of PrEP to 3800 individuals at risk of acquiring HIV in Victoria. This study will provide important information for the broader implementation of PrEP planning upon listing of the tenofovir-emtricitabine on the national subsidized list of medicines. The study is registered on the Australian New Zealand Clinical Trials Registry (ACTRN12616001215415)

## Introduction

In Australia, HIV is largely concentrated among gay, bisexual and other men who have sex with men (GBM). Almost three quarters of annual notifications are among men reporting male to male sex as their exposure to HIV, with other exposures such as injecting drug use, heterosexual sex and being born in a high prevalence country being less commonly reported (1). In 2015 there were 1025 new HIV diagnoses in Australia and there were an estimated 25,313 people living with HIV (PLWH) (estimated prevalence of 0.1%) (1, 2). Despite significant investment in, and uptake of, HIV prevention activities, the annual number of HIV notifications has remained stable over the last four years (1).

HIV pre-exposure prophylaxis (PrEP) is the newest tool in the HIV prevention toolkit. PrEP is the use of tenofovir and emtricitabine on a daily or on demand schedule to prevent HIV transmission (3, 4). There is substantial evidence of the safety, efficacy and effectiveness of PrEP at reducing HIV transmission in GBM, trans-women, heterosexual men and women and people who inject drugs (3, 5–11). The efficacy of PrEP is directly correlated with medication adherence (12, 13) with recent open label trials reporting high adherence and greater reduction in risk of HIV acquisition compared to earlier, blinded studies (13, 14). While PrEP is highly effective at reducing HIV transmission it confers no protective effect against sexually transmissible infections (STIs) with the exception of HSV2 infections (15, 16). Concerns have been raised that PrEP use would increase condomless peno-anal or peno-vaginal sex, number of sex partners and in turn incidence of STIs, however there is mixed evidence of such changes occurring (11, 14, 17). In 2015 the World Health Organisation recommended PrEP as an HIV prevention option for all key populations (18). As at November 2017, PrEP is approved for use by the regulatory authorities of at least 20 countries, including Australia. Although approximately 30 countries currently run or plan to implement PrEP demonstration projects (19), there are as yet no publications which confirm the population level impact of PrEP.

Australian PrEP guidelines are based on individuals' risk of HIV acquisition. PrEP is recommended for people reporting high or moderate risk for acquiring HIV through male to male sex, heterosexual sex, and/or injecting drug use. Recent changes to the guidelines also permit clinicians' discretionary prescription of PrEP (20).

PrEP is currently registered for use in Australia, and will be listed on the national list of medicines [the Pharmaceutical Benefits Scheme (PBS)] on 01 April 2018. In 2016, three pharmaceutical companies had their co-formulated tenofovir and emtricitabine products approved by Australia's Therapeutic Goods Administration (TGA) for use in HIV prevention (21–23). In Australia, medicines listed on the PBS cost $39 AUD or $7 AUD for person eligible for concession (24) per dispensed medication, but because PrEP is not currently subsidized through the PBS obtaining PrEP via a private prescription incurs costs up to $900 AUD per month (25, 26). Generic PrEP can be imported from overseas through the TGA personal importation scheme, wherein registered medical practitioners provide individuals with clinical monitoring and 3-monthly prescriptions for PrEP (27). The cost of personally importing PrEP ranges between $70–230 USD (approximately $90–300 AUD) for a 3 month supply (28, 29); delays in customs and shipping have been reported (30). Finally, PrEP can be accessed through jurisdiction-level PrEP implementation projects, which either provide participants free study medication, or require that participants pay costs commensurate with current PBS prices. Six of the eight Australian jurisdictions offer PrEP via population intervention studies, with the size and number of studies increasing significantly since the first PrEP demonstration project, VicPrEP, began enrolment in Victoria in June 2014 (Table 1) (17, 31–36). VicPrEP demonstrated that PrEP could be successfully prescribed and managed in general practice in Victoria, providing preliminary evidence for the current, larger demonstration study, PrEPX (17).

**Table 1:** PrEPX study timeline, 2013–2019.

**Phase**	**Date**	**Activity**
Pre-PrEPX	2013–2014	Community advocacy for PrEP access and forums held by VAC to discuss PrEP, PrEP stigma and future clinical trials.
	Jun 2014–Dec 2016	VicPrEP study−30 month demonstration project enrolled 114 participants across four sites in Melbourne.
	Jun–Aug 2015	PrEP'D for Change and PrEPaccessNOW formed. General practitioners developed guidelines for prescribing off-label PrEP.
	Dec 2015	Victorian PrEP Accord was signed. Signatories include VAC, LPV, PrEP'DforChange, PrEPaccessNOW, time4PrEp, VicPrEP.
PrEPX planning	Sep-Dec 2015	Approached clinics and community pharmacies to participate in the PrEPX study. Approached peak organizations including VAC, LPV, VACCHO, Centre for Culture, Ethnicity and Health.
	29 Jan 2016	Funding announced by the Victorian DHHS, Alfred Health and VAC for 2,600 places in PrEPX study.
	30 Jan 2016	Study registration opened using REDCap on the Alfred hospital website. Data collected: name, contact details, preferred clinic to attend for PrEPX, whether participant was on PrEP.
	22 Jul 2016	2049 people registered interest on online formClinics provided with partially de-identified list from registration form to enable workforce planning when study openedRegistered individuals emailed about study opening dates and booking information for their preferred clinic.
PrEPX study period	26 Jul 2016	PrEPX enrolment began (wave one of study enrolment) 2600 places opened, 150 of these reserved for priority populations including people who inject drugs and/or people in serodiscordant heterosexual partnerships with high or medium risk for HIV acquisition as per PrEP prescribing guidelines (Table 2).
	14 Oct 2016	PrEPX study neared enrolment capacityStudy enrolment stopped at 2200 participants to allow for people who had booked an appointment and not yet attended to join the study, with other places reserved for priority populations.First waitlist was opened, pending further funding.
	17 Jan 2017	Additional 600 places in PrEPX announced, funded by VAC.
	19 Jan 2017	Additional places released (wave two of study enrolment)All 575 people registered on the waitlist were provided a study place. Clinics were provided a list of participants as per original PrEP enrolment.
	20 Jan 2017	Waitlist was reopened.
	28 Mar 2017	Additional 600 places announced, funded by Victorian DHHS.
	29 Mar 2017	Additional places released (wave three study enrolment)All 617 people registered on the waitlist were provided a study place Clinics were provided list of participants as per original PrEP enrolment.
	30 Mar 2017	Waitlist was reopenedWaitlist remains open November 27th 2017.
	30 June 2019	PrEP-X study estimated end date.

*VicPrEP, Victorian PrEP demonstration study; PrEPX, Victorian PrEP population level study; PrEPX-SA, expansion of PrEPX study to South Australia; VAC, Victorian AIDS Council, LPV, Living Positive Victoria; VACCHO, Victorian Aboriginal Community Controlled Health Organisation Inc.; DHHS, Department of Health and Human Services*.

### Rationale for this study

PrEP randomized controlled trials and PrEP demonstration projects have shown that the use of tenofovir and emtricitabine is safe, highly efficacious and effective in the prevention of HIV infection (3, 6, 9, 11). Based on this current knowledge of PrEP we are introducing this HIV intervention tool at a population level in Victoria to people at high risk of HIV infection to help reduce new HIV infections in the State.

The VicPrEP study showed that during a period of 12 months of PrEP use, adherence to drug schedule was high. (17). In some studies, including VicPrEP, STIs have increased and condom use has declined (11, 17). However interpretation of these findings is not straightforward and requires further follow-up combined with the planning and implementation of innovative STI prevention strategies. Furthermore, individuals who receive PrEP attend their healthcare practitioners on a quarterly basis, which may afford individuals the opportunity to enhance other aspects of their health including blood pressure management, assistance with managing depression, drug and alcohol use and vaccinations.

### Study aims

In Victoria, three quarters of new HIV diagnoses are among GBM and the knowledge of, interest and willingness to use PrEP is high amongst GBM at higher risk of HIV acquisition (37). We hypothesize that the provision of PrEP to individuals at high risk of HIV would be both justified and feasible and could achieve a population level reduction in HIV incidence. The primary aim of this study is to determine if the provision of PrEP for 36 months to 2,600 people at high risk of HIV infection in Victoria will result in a 30% decline in new HIV infections in GBM and a 25% state wide decline in new HIV infections.

The study's secondary aims are to explore:

- Baseline demographics, behavior, HIV risk factors, prevalence of STIs and blood borne viruses among GBM presenting for PrEP.
- Attitudes to PrEP, reasons for taking PrEP and attitudes around sexual behavior since the availability of PrEP.
- PrEP adherence.
- Changes in sexual behavior and STI rates among PrEP users.
- Use of non-occupation post exposure prophylaxis (NPEP) in Victoria.
- The capacity of study clinics to prescribe PrEP.
- Any ancillary health outcomes associated with PrEPX participation.

## Methods

### Study design

PrEPX is a prospective, population-level intervention study, designed to emulate “real world” conditions to reflect the likely clinical scenario were tenofovir and emtricitabine to become subsided by the PBS for use as HIV PrEP in Australia. People interested in participating in PrEPX may register their interest in the registration or waitlists or directly approach participating clinics to enquire about the study. Participating clinicians may recommend PrEP, and participation in PrEPX, to their clients. Participants will be provided with daily co-formulated generic tenofovir with emtricitabine purchased from Mylan Pharmaceuticals, Australian Sponsor- Alphapharm (38).

The investigators approached community organizations in Victoria representing people who are gay and bisexual, trans and gender diverse, Aboriginal and/or Torres Strait Islanders, and culturally and linguistically diverse to gain their input on the PrEPX study design and suitability for their constituents, prior to obtaining study funding. Organizations were offered financial support to develop pathways to facilitate their target populations' participation in PrEPX. All the community organizations approached agreed to engage with and support PrEPX (Table 1).

### Study setting

The study is set in Victoria, the second most populous Australian state with a population of 6.2 million people and about 45,000 GBM (2, 39). In 2015, there were 286 new HIV diagnoses in Victoria of which 148 were defined as newly acquired [evidence of diagnosis within 12 months of infection (40)], with diagnosis rates of 4.8 and 2.0 per 100,000 population, respectively (1). In metropolitan Melbourne (the capital of Victoria), where approximately 80% of people newly diagnosed with HIV in Victoria reside (41), PLWH and GBM are cared for by three types of clinics: a small number of general practice clinics that have high caseloads of PLWH and GBM, state-funded sexual health services, and infectious disease (ID) clinics at teaching hospitals. The Alfred Hospital provides a state wide HIV service. These three types of clinics are staffed by general practitioners, sexual health clinicians and infectious disease clinicians who are licensed to prescribe antiretroviral medications on the PBS. Since 2013, the PBS has provided ARVs to PLWH at any CD4 cell count (previously restricted to a CD4 cell count below 500 cells/ml). PrEPX study sites include GBM specialist GP clinics, state-funded sexual health service, the Alfred hospital HIV service, shared care and outreach study sites (Table 1).

### Engagement of clinics, pharmacies and community organizations

Prior to seeking funding for PrEPX, the investigators approached seven clinical services to determine their interest and capacity to undertake the PrEPX study. The study offered either a $100 AUD payment per participant enrolled, or a part-time nurse to support enrolment of study participants. All clinical sites approached by investigators (*n* = 7) agreed to participate in the study and then received site initiation and training by an investigator and/ or a clinical trial nurse.

In Australia clinical trial medications are conventionally free of charge and are dispensed by hospital-based pharmacies. However, to mimic real world conditions, PrEPX participants can have their PrEP medication dispensed by community pharmacies located close to the study clinics. Participants are required to pay a co-payment to simulate the co-payment costs that Australian residents pay for medications listed on the PBS (26). The investigators approached five community pharmacies to determine if they would dispense PrEPX study medication. All community pharmacies approached by investigators agreed to participate in the study and received appropriate facilities and training to conform with good clinical research practice.

### Shared care and outreach study sites

The PrEPX shared care model is based on existing models of clinical shared care in Victoria. These models ensure individuals are able to access services across outer metropolitan Melbourne, regional and rural Victoria. The shared care model aims to improve geographic access, equity and convenience and the model was used to provide PrEP sites outside of metropolitan Melbourne.

Within this shared care model the participant is enrolled as a study participant at the Alfred Hospital while receiving care with their general practitioner at a remote site (remote GP). The PrEPX investigator (based at the Alfred hospital) is responsible for confirming study eligibility, for enrolling the participant, providing clinical monitoring, PrEPX study drug prescription and oversight of the shared care remote GP. Test results are maintained at both the remote GP and Alfred Hospital PrEPX sites; results are discussed between practitioners (PrEPX investigator and shared care site GP) as required. Study drug can be dispensed by a participating PrEPX community pharmacy site or the hospital pharmacy can dispense and send the study drug to the participant via registered mail. For participants receiving study drug at the baseline visit via mail, the investigator provides a follow-up phone call to the participant, reaffirming dispensing information and receipt of study drug.

Within the outreach clinic model a clinician from the Alfred Hospital attends a rural or regional clinic in Victoria. Study participants are enrolled at the remote clinic into the PrEPX study as an Alfred Hospital patient and assigned an Alfred Hospital unique reference number.

Details of the participant's enrolment are entered into the participant's local medical records at the outreach clinic. The Alfred Hospital PrEPX clinician will have access to the study participants medical records at the outreach clinic. Pathology tests are collected at a local Pathology Service provider and the results are sent to the Alfred Hospital and to the outreach clinic. Follow-up care is provided by the local healthcare provider. Study drug is dispensed and sent to the participant via registered mail from the Alfred Hospital Clinical Trials pharmacy.

### Study funding

PrEPX received funding from the Victorian Department of Health and Human Services (DHHS) on 29 January 2016 for 2,600 study places. An additional 600 places were funded by the Victorian AIDS Council (announced on 19 January 2017) and a further 600 places were funded by the Victorian DHHS (announced on 28 March 2017). Hence a total of 3,800 participants will be enrolled into the PrEPX study. Waitlists were established in anticipation of these two funding injections (Table 1).

### Study participants

#### Inclusion criteria

Eligible participants must be HIV-negative at their baseline PrEPX appointment, aged 18 years and over, have an estimated glomerular filtration (eGFR) rate of >60 mL/min/ 1.73 m^2^ and meet the behavioral eligibility criteria of the 2015 ASHM PrEP Guidelines (42). Eligible participants are individuals reporting male to male sex, injecting drug use and/or serodiscordant heterosexual sex with high or medium risk for HIV acquisition (Table 2) (42). Individuals not meeting these behavioral eligibility criteria can be enrolled at the clinician's discretion, if the clinician is of the opinion that the patient would benefit from being on PrEP (Table 2).

**Table 2:** Eligibility criteria for PrEPX Study.

	**Gay, bisexual and transgender people**	**Heterosexual and transgender people**	**People who inject drugs**
Predicted risk of the study participant in the next 3 months	Likely to have multiple events of condomless anal intercourse (CLAI), with or without sharing intravenous drug use equipment	Likely to have multiple events of condomless anal or vaginal intercourse, with or without sharing intravenous drug use equipment	Likely to have multiple events of sharing needles or injecting equipment with an HIV positive individual or a homosexually active man and has inadequate access to safe injecting equipment
**AND**			
Reported risk in the past 3 months (unless other timeframe stated)	Is a regular sexual partner of an HIV-positive male partner with whom condoms were not consistently used (HIV positive partner is not on treatment and/or has detectable HIV viral load)	Is a regular sexual partner of an HIV-positive man or woman with whom condoms were not consistently used in the last 3 months (HIV positive partner is not on treatment and/or has detectable viral load)	
	At least one episode of receptive CLAI with any casual HIV-positive male partner or a male partner of unknown HIV status		
	A diagnosis of rectal gonorrhoeae, chlamydia and/or syphilis during the last 3 months or at screening		
	Has used methamphetamine		
	Has had more than one episode of anal intercourse in the last 3 months when proper condom use was not achieved (e.g., condoms slipped off or broke)		
	Is uncircumcised and reports more than one episode of insertive CLAI in the last 3 months where the serostatus of their partner was not known, or the partner was HIV positive and not on antiretroviral treatment		
			Has been sharing needles or injecting equipment with an HIV positive individual or with a homosexually active man
**Clinicians' discretion**			
All people who do not report any of the above risk factors should still be considered at risk of HIV infection and evaluated on a case-by-case basis.			

*Adapted from National prescribing guidelines (20)*.

#### Exclusion criteria

Participants are excluded if they are HIV-positive as confirmed by HIV antibody/antigen and western blot testing; have signs and/or symptoms of acute HIV infection; are not eligible for Australia's universal healthcare system Medicare (43); are unwilling to provide consent to follow-up; have an eGFR of <60 mL/ min/ 1.73 m^2^; currently use medications which interact with PrEP medication; have concomitant participation in another clinical trial using investigational agents, or have any other condition that, based on the opinion of the treating clinician, would make participation unsafe (Table 3).

**Table 3:** Study procedure schedule of assessment.

	**Baseline**	**3 m**	**6 m**	**9 m**	**12 m**	**15 m**	**18 m**	**24 m**
Informed consent sought	X							
HIV Ab/Ag test	X	X	X	X	X	X	X	X
Syphilis serology	X	X	X	X	X	X	X	X
CT/NG testing	X	X	X	X	X	X	X	X
Pregnancy test ^*^	X	X	X	X	X	X	X	X
HBsAg & HBsAb HBV vaccine status	X							
HCV Ab	X				X			X
Serum creatinine & eGFR	X		X		X		X	X
Enrolment Survey	X							
Behavioral survey	X	X	X	X	X	X	X	X
Online in depth survey	X		X		X		X	X
In depth interview	X							X
Clinic level survey	X				X			X

* *Heterosexually active females only (participants reporting risk for pregnancy only)*.

*Ab/Ag, antibody/antigen; CT, Chlamydia trachomatis; NG, Neisseria gonorrhoeae; HBsAG, hepatitis B virus surface antigen; HBsAb, hepatitis B virus surface antibody; HCV, hepatitis C virus; eGFR, estimated glomerular filtration rate*.

### Study visits

At baseline, written informed consent is obtained and clinicians complete and submit an online enrolment survey through the Research Electronic Data Capture system (REDCap) (44). REDCap immediately generates the participant's study number and sends it via email to the participant along with the contact details of study personnel (Table 3). Clinicians are advised to provide a prescription for 3 months of PrEP at the baseline visit unless they are concerned the participant is HIV positive, or has renal impairment. Follow-up study visits occur every 3 months. At each study visit participants undergo scheduled HIV and STI testing and received a 3-month PrEP prescription (Table 3). Renal monitoring is performed 6-monthly, or more frequently as required.

Each participant's study visit attracts a Medicare rebate for consultations and pathology requests because regular clinical, HIV, STI and laboratory monitoring are standard-of-care for GBM at high risk of HIV infection in Australia (45, 46). In addition, participants who enrol at one of the participating general practice clinics will be billed for their visits in accordance with the existing billing policies of those clinics.

Participants attending one of the seven clinics will be able to attend a linked community pharmacy and will be required to pay the PBS co-payment ($7 or $39 AUD) as per usual practice in Australia. Participants attending Melbourne Sexual Health Centre or the Alfred Hospital will be invoiced for the co-payment.

### Data collection

#### Registration and waitlist data collection

When study funding was announced in January 2016, a database (study registry) was established so that individuals could register their interest in the study thereby assisting the study team's plans for study implementation (Table 1). The register collects participants' contact details, previous PrEP use and which study site participants would prefer to attend.

A few days prior to commencement of study enrolment, registered individuals were emailed information on how to book a PrEPX appointment at their preferred study site and information on whether co-payment would likely be required by the clinic at study enrolment. Clinics were provided with a list of individuals planning to attend their clinic, including contact details, if currently using PrEP and if currently a client at that clinic.

The study opened on 26 July 2016 and data collection among enrolled participants is scheduled until July 2019.

As enrolment neared capacity (*n* = 2,600) a waitlist register was established so individuals could register their interest if further places became available. The waitlist register collected the same variables as the initial registry of interest. Consistent with the protocol at study opening, with each release of additional study places registered individuals were emailed details of their preferred study site and clinics were emailed information on their potential PrEPX participants.

#### Participants' baseline and follow-up data collection

The laboratory investigations and surveys that participants undergo at baseline are outlined in Tables 3, **4**. All study participants are evaluated with baseline laboratory tests and the baseline enrolment form survey (Tables 3, 4, respectively). Participants will be offered hepatitis B vaccination as required. Further surveys include a self-reported behavioral survey completed by participants attending five study clinics participating in the Australian Collaboration for Coordinated Enhanced Sentinel Surveillance of Blood Borne Viruses and Sexually Transmitted Infections system (ACCESS, described below), the ACCESS behavioral survey. An in-depth, online survey performed at baseline then 6-monthly thereafter was completed by 1,200 participants at enrolment. This survey provides more detailed information on sexual behavior, adherence, attitudes to PrEP and acceptability of the study. An in-depth, face-to-face interview at baseline and month 21 was completed by up to 30 participants, purposively selected from those completing the in-depth online survey (Table 4).

**Table 4:** Data fields collected in each PrEPX survey.

	**Enrolment form Survey**	**ACCESS behavioral survey**	**In-depth online survey**
**DEMOGRAPHICS**			
Age	X	X	X
Country of birth	X	X	X
Year of arrival	X	X	
Aboriginal and/or Torres Strait islander	X (since 2017)	X	X
Gender identity	X	X	X
Sex assigned at birth	X	X	
Sexuality	X		
Residential postcode			X
Education level			X
**HIV/STI TESTING HISTORY**			
Time since last HIV test	X	X	X
Time since last STI test	X		X
Place of last HIV test		X	
Reason for HIV test		X	
**Sexual Behaviors and Risks**			
Gender and number of sex partners		X	X
Condom use regular and casual partners		X	X
Regular partners/sex/HIV status			X
Other risk reduction with casual partners		X	X
Group sex		X	
Drug use		X	X
Injecting drug use	X	X	X
Sex work		X	X
**PREVIOUS PEP/PrEP**			
Previous PEP use	X		X
Previous PrEP use	X	X	X
Method of acquiring PrEP	X		X
**PrEP ELIGIBILITY ASSESSMENT**			
GBM risk assessment	X		
Hetero risk assessment	X		
Transgender risk assessment	X		
IDU risk assessment	X		
Clinician discretion	X		
**CLINICAL PRESENTATION**			
Acute HIV or STI symptoms	X		
Past medical history	X		
Current medication	X		
**CLINICAL RESULTS**			
eGFR, HIV, HBV, HCV, Pregnancy	X		
**Beliefs about HIV**			X
**Attitudes to PrEP**			X
**Attitudes to HIV treatment and prevention**			X
**Condom use & opinions**			X
**STIs**			X
**Clinic Visit Experience**			X

PrEPX utilizes the ACCESS system to collect HIV, STI and other test information. ACCESS includes primary care, sexual health and pathology services with data primarily collected through the GRHANITE^TM^ data extraction software (47). GRHANITE^TM^ interfaces with clinic patient management systems to extract patient test results, demographics, and prescriptions and with pathology service data systems to extract test results and demographics (47). Test results routinely collected as part of ACCESS include HIV, *Chlamydia trachomatis* (CT), *Neisseria gonorrhoeae* (NG), *Treponema pallidum* (syphilis), hepatitis B virus and, hepatitis C virus. Tests additionally collected for PrEPX include urine protein: creatinine ratio, serum creatinine, and eGFR. GRHANITE^TM^ will be used also to extract data to evaluate whether enrolment in PrEPX is associated with an increase in ancillary health outcome measures including diagnosis and treatment of hypertension, depression and other common medical conditions.

GRHANITE^TM^ data extraction software removes all patient identifying details before data extraction from the clinics or pathology services. Patients are assigned a unique identifier, which allows longitudinal analysis of a patient within a clinic. PrEPX participants are re-identified within ACCESS study sites through matching a numerical identifier, year of birth and enrolment clinic within the enrolment survey and the ACCESS dataset. De-identified test results are stored at the Burnet Institute.

#### Other study data

Clinic staff at three of the four Victorian primary care clinics, two sexual health clinics, and the hospital clinic have been invited to complete a survey about characteristics of their clinic, their PrEP patient load and clinic level challenges experienced during the study at baseline, months 12 and 24.

Pharmacy data including drug accountability logs and participant record logs are manually collected from each participating site.

Victorian HIV diagnosis data will be collected from the Victorian Department of Health and Human Services (DHHS). It is a legal requirement that all newly diagnosed cases of HIV are notified to the DHHS. Notifications include test result data from the laboratory and an enhanced surveillance form from the diagnosing doctor that includes demographics, test history and exposure to HIV. Non-identifiable line listed data will be collected from the Victorian DHHS including the diagnosis date, country of birth, age, and exposure to HIV for all newly diagnosed cases in Victoria between 2013-2019.

### Sample size estimates

We estimated that there are 300 new HIV infections per year in Victoria based on back-calculation methods applied to surveillance data and a modeling framework. With high and stable testing rates and HIV notification trends in Australia, these estimates have demonstrated strong concordance with actual trends in new infections (1, 48, 49). Approximately 75% of new HIV infections in Victoria occur in GBM, providing an estimate that 225 new infections occur in GBM in Victoria, annually.

We powered the study to avert 68 new HIV infections annually among GBM, which equates to a 30% reduction in new HIV infections in GBM in Victoria. We consider that this would represent a meaningful population-level prevention outcome from scaling up PrEP. Power calculations were based on a HIV incidence estimate of 2.78 per 100 person years (PY) from high risk GBM (consistent with risk criteria guidelines for PrEP) in the Health in Men (HIM) cohort study (50). The HIM study measured HIV incidence among GBM in Sydney in the early-to-mid 2000s (50). There are not adequate, contemporary Victorian data to determine HIV infection risk for GBM, but participants in HIM were not dissimilar in characteristics nor in epidemiological context to GBM in Victoria and Wilson et. al. has shown that the per-capita transmission rate has remained relatively stable since that period (51). We estimated that the number of GBM at risk of HIV in Victoria was 28,846 (225/0.78 per 100PY). With an estimated 24% classified as high risk [as per the HIM study (50)] and hence targeted for study participation, our target population size was therefore estimated as 6,923 (28,846 × 0.24). We estimated that a sample size of 2,600 participants (37% of high risk GBM) was required to achieve the 30% annual HIV infection reduction outlined above. Achieving this PrEP coverage was considered feasible given recent survey data showing that 32% of GBM classified as having high HIV acquisition risk were both willing and eligible to use PrEP (37).

### Statistical analyses

The study will determine if the provision of PrEP to 2,600 people at high risk of HIV infection in Victoria results in a 30% decline in new HIV infections among GBM over the 36 months of the study. In addition, the study will monitor the rate of new HIV infections in Victoria for a period of 36 months after the PrEPX study commences. Analyses of changes in the rates of HIV infection will be undertaken using Mann-Whitney U and chi-square tests to determine whether there has been a significant decline in new HIV infections during this 36-month period, compared to the 36 months prior to PrEPX commencing.

We will undertake further analyses to determine whether or not other factors such as an increase in background HIV and STI testing rates during the PrEPX study period and an increase in the background rate of HIV virological suppression of HIV positive people living in Victoria during the PrEPX study period may have contributed to any decrease in new HIV infections. These analyses will be undertaken using Cox proportional and logistic regression analyses to calculate hazard or rate ratios and odds ratios, respectively, with 95% confidence intervals. To determine the factors associated with incident HIV infection among trial participants, we will explore demographic and behavioral risk factors.

To answer the study secondary aims a range of datasets and analyses will be employed, as required. The enrolment survey, ACCESS behavioral survey, and ACCESS test data will be used to explore baseline demographics, behavior, HIV risk factors, prevalence of STIs and blood borne viruses among GBM presenting for PrEP, changes in behavior and STI rates among PrEP users and ancillary health benefits among PrEP users. The in-depth online survey and interviews will be used to explore attitudes to PrEP, reasons for taking PrEP and attitudes around sexual behavior since the availability of PrEP. We will use ACCESS test data, pharmacy data and in-depth online surveys to explore PrEP adherence. Monthly aggregate data from the state NPEP service will be descriptively analyzed to explore state wide use of clinic survey and ACCESS test data will be used to explore the capacity of study clinics to prescribe PrEP.

All statistical analyses will be performed using Stata version 14 (StataCorp LP, College Station, Texas, USA) with a significance cut off of *p* = 0.05.

### Ethics

Written informed consent is obtained from all study participants. The PrePX study was approved by the Alfred Health Human Research and Ethics Committee (HREC100/16) and registered on the Australian New Zealand Clinical Trials Registry (ACTRN12616001215415).

## Discussion

PrEP is a highly effective HIV prevention tool and may contribute to a meaningful decline in HIV transmission in Australia. PrEPX will deliver PrEP to 3800 individuals at high risk of HIV acquisition in Victoria. The PrEPX study will explore if the provision of PrEP contributes to a decline in HIV incidence, is associated with a change in participant behaviors, a change in the incidence of STIs and the capacity of clinics to provide PrEP on a large scale. This study design closely emulates a real world scenario and is likely to provide important data to guide PrEP implementation once PrEP become listed on the PBS in Australia.

The design of PrEPX, is a key strength of the study. Community groups have representatives as co-investigators on the study and provide information to their communities and to the study team, contributing to iterative refinement of the study. Consultation with clinics allowed clinics to plan for study enrolment and to provide feedback to the study team when they reached capacity. Electronic data collection, including study enrolment, behavioral data and test data collection decrease the clinical trial burden placed on clinics and with the exception of enrolment, utilize the existing ACCESS sentinel surveillance system at most clinic sites. This design provides long term, high quality clinical and behavioral data among GBM testing for HIV and STIs, allowing for population level estimates of the impact of PrEP while maintaining standard practice at participating sites. At the drug dispensing level the involvement of community pharmacies and the requirement for participants to pay for study drug in accordance with government approved dispensing fees following PBS listing, will provide important information on affordability in a real world setting and will further allow trial participants to better plan for ongoing costs of taking PrEP once approved on the PBS.

There are a number of limitations in this study. While efforts were made to emulate real world setting the exclusion criteria, limited number of study spaces, and compensation of clinics for each enrollee introduce aspects to the study that will not be present when PrEP is listed on the PBS. Also, while there is significant data collected in this study, the restriction of behavioral and test data is only collected from sites participating in the ACCESS surveillance system, however we anticipate that the vast majority of clients will be enrolled at these sites. Finally, HIV diagnoses are being used as a proxy for HIV incidence and may not accurately reflect incidence. However, we aim to triangulate HIV notification in Victoria, with diagnoses and testing at ACCESS surveillance sites to improve estimates of the impact of PrEPX.

PrEPX will provide important evidence on an approach that emulates the real world when implementing PrEP on a large scale at a time when much of the world is transitioning to the use of this new HIV prevention tool. The PrEPX study design will allow researchers to determine if provision of PrEP to people at risk of HIV in Victoria reduces HIV transmission. In addition, it will provide important information on behavioral change and STI rates, attitudes toward PrEP, ancillary health benefits of quarterly clinical visits and the health service requirements for providing PrEP on a large scale. PrEPX will deliver implementation and population level results that will be transferrable to other Australian jurisdictions and international settings.

## Author contributions

AM, BP, CF, SR, LL, CoB, DW, JL, DM, VC, NR, JWillcox, CC, JAr, BT, MP, GS, CW, JM, KB, AC, BA, JWiggins, JK, OV, MW, AP, DG, ChB, JdeW, JH, SW, RG, and EW contributed to the design of the project. KR, MS, JAs, CE-H, LN, DM, VC, NR, JWillcox, CC, JAr, BT, MP, G-FS, and EW contributed to data collection. KR, AM, MS, CF, JAs, VC, JH, and EW contributed to manuscript preparation. All authors reviewed and approved the final draft of the manuscript.

### Conflict of interest statement

The authors declare that the research was conducted in the absence of any commercial or financial relationships that could be construed as a potential conflict of interest. The reviewer CE and handling Editor declared their shared affiliation.
